# Effects of high-intensity focused ultrasound combined with levonorgestrel-releasing intrauterine system on patients with adenomyosis

**DOI:** 10.1038/s41598-023-37096-y

**Published:** 2023-06-19

**Authors:** Yuru Cai, Yanan Sun, Feng Xu, Yunzhe Wu, Chunfeng Ren, Xiaohong Hao, Bulang Gao, Qinying Cao

**Affiliations:** 1 Department of Obstetrics and Gynecology, Shijiazhuang People’s Hospital, 365 South Jianhua Street, Shijiazhuang, 050030 Hebei Province China; 2 Department of Laboratory Analysis, The First Affiliated Hospital of Zhengzhou University, 1 Longhu Middle Ring Road, Zhengzhou, 450018 Henan Province China; 3 Shijiazhuang People’s Hospital, 365 South Jianhua Street, Shijiazhuang, 050030 Hebei Province China

**Keywords:** Diseases, Health care, Medical research

## Abstract

It is very important to treat adenomyosis which may cause infertility, menorrhagia, and dysmenorrhea for women at the reproductive age. High-intensity focused ultrasound (HIFU) is effective in destroying target tumor tissues without damaging the path of the ultrasound beam and surrounding normal tissues. The levonorgestrel-releasing intrauterine system (LN-IUS) is a medical system which is inserted into the uterine to provide medicinal treatment for temporary control of the symptoms caused by adenomyosis. This study was to investigate the effect of HIFU combined with the LN-IUS on adenomyosis. In the HIFU treatment, the parameters of the ultrasound were transmission frequency 0.8 MHz and input power 50–400 W (350 ± 30), and the temperature in the target tissue under these conditions would reach 60–100 °C (85 °C ± 6.3 °C). Size reduction and blood flow signal decrease were used to assess the effect of combined treatment. In this study, 131 patients with adenomyosis treated with HIFU combined with LN-IUS were retrospectively enrolled. The clinical and follow-up data were analyzed. After treatment, the volume of the uterine lesion was significantly decreased with an effective rate of 72.1%, and the adenomyosis blood flow signals were significantly reduced, with an effective rate of 71.3%. At six months, the menstrual cycle was significantly (*P* < 0.05) decreased from 31.4 ± 3.5 days before treatment to 28.6 ± 1.9 days, the menstrual period was significantly shortened from 7.9 ± 1.2 days before HIFU to 6.5 ± 1.3 days, and the menstrual volume was significantly (*P* < 0.05) decreased from 100 to 49% ± 13%. The serum hemoglobin significantly (*P* < 0.05) increased from 90.8 ± 6.2 g/L before treatment to 121.6 ± 10.8 g/L at six months for patients with anemia. Among seventy-two (92.3%) patients who finished the six-month follow-up, sixty-five (90.3%) patients had the dysmenorrhea completely relieved, and the other seven (9.7%) patients had only slight dysmenorrhea which did not affect their daily life. Adverse events occurred in 24 (18.3%) patients without causing severe consequences, including skin burns in two (1.5%) patients, skin swelling in four (3.1%), mild lower abdominal pain and low fever in 15 (11.5%), and subcutaneous induration in three (2.3%). Six months after treatment, no other serious side effects occurred in any patients with follow-up. In conclusions, the use of high-intensity focused ultrasound combined with the levonorgestrel-releasing intrauterine system for the treatment of adenomyosis is safe and effective even though the long-term effect remains to be confirmed.

## Introduction

Uterine adenomyosis is a common benign pathological condition for women during the reproductive age, with an incidence ranging from 20 to 40%^1–5^. As a common gynecological disease, adenomyosis is characterized by presence of ectopic endometrial glands and stroma in the myometrium of women at the reproductive age^6–8^. This condition may severely affect the fertility and daily life of women with cyclic menstruation symptoms including infertility, menorrhagia, and dysmenorrhea. Treatment approaches for this condition include medication, surgery, radiofrequency ablation, uterine arterial embolization (UAE), and high-intensity focused ultrasound (HIFU). Medications, including levonorgestrel-releasing intrauterine system (LN-IUS), gonadotropin-releasing hormone agonist and oral contraceptives, may provide temporary control of the symptoms but may be futile in resistant cases^2^. Conservative surgical treatment like local excision of the lesion may not be effective to preserve fertility because of ill-defined myometrial-endometrial boundaries^7,9^. Moreover, fibrotic scars and sutures in adjacent healthy tissues may negatively affect fertility in the future. Hysteroscopic excision may be effective for superficial lesions but may result in residuals for deep-located lesions. UAE is minimally invasive by blocking blood vessels to the lesion, but may have adverse events on ovarian function and fertility^5,10,11^. Moreover, UAE may not block all supplying vessels to the lesion, resulting in residual lesions and symptoms.

HIFU is a new technology for non-invasive treatment of tumors in vivo, which can effectively destroy the target tissue without damaging the path of the ultrasound beam and surrounding normal tissue^1–5,7^. Ultrasound beam can penetrate into deep tissues and cause protein denaturation and irreversible cellular death via coagulative necrosis in the target tissue using thermal energy^12–16^, which is one of the primary mechanism of HIFU application in medicine^7^. The second mechanism is an indirect effect of HIFU through inducing necrosis or embolism of nutrient vessels of the target tissue to obstruct the vascular supply and inhibit the growth^7^. As a complex phenomenon, cavitation refers to the production of bubbles in fluids and the action of ultrasound energy on these bubbles. The cavitation effect of HIFU can cause intense disruption of tissue structures^16^. By these mechanisms, HIFU has achieved good effects in the treatment of benign and malignant tumors such as prostate, breast, liver, pancreas, kidney, and bone^6,17–24^. Its thermal ablation effect has little trauma to the body^1,2^. By accurately focusing the ultrasound energy in vivo, it can produce an instantaneous high temperature effect (60–100 ℃) and a cavitation effect in the tumor^12–16^, which can destroy the tumor tissue and achieve the purpose of non-surgical treatment. It was hypothesized that HIFU combined with the LN-IUS would be able to improve the treatment effect of either one approach alone. This study was consequently performed to investigate the effect of HIFU combined with the LN-IUS in the treatment of adenomyosis through analyzing the effect on the uterine size, blood flow signal, and clinical presentations before and after treatment.

## Materials and methods

### Subjects

This retrospective longitudinal one-center study was approved by the ethics committee of Shijiazhuang People’s Hospital, and all patients had signed the informed consent to participate. All methods were performed in accordance with the relevant guidelines and regulations. Between November 2011 and December 2020, patients who had been diagnosed as adenomyosis using ultrasound and magnetic resonance imaging (MRI) and treated with HIFU combined with LN-IUS were enrolled. The inclusion criteria were patients with adenomyosis, treated with both HIFU and LN-IUS during the non-menstrual period, control of pelvic inflammation, removal of the intrauterine contraceptive device, and no vaginal bleeding. The exclusion criteria were location of the lesion in the cervix, patients with suspected sarcoma, cervical lesions, endometrial cancer and other malignant tumors, and patients with severe heart, brain and lung diseases.

### Equipment

The JC-200 focused ultrasound tumor treatment system developed by Chongqing Haifu Medical Technology Co. Ltd (Chongqing, China, http://www.haifumedical.com/) was adopted. The whole system is mainly composed of a HIFU treatment system which produces high-energy ultrasound, a water management system, a diagnostic ultrasound system for real-time monitoring of the tumor texture, and a power source. The whole system produces high-energy ultrasound focuses on a point in the tumor in vivo through the skin, takes degassed water as the medium, and generates an instantaneous high temperature (60–100 ℃) in the lesion for treatment^12–16^, resulting in tissue coagulative necrosis to achieve the purpose of treatment.

### HIFU procedures

Before treatment, routine examination was performed of blood, urine, stool, liver and kidney function, and electrocardiogram. Routine ultrasound examination was carried out for the position, shape and size of the uterus, size of uterine lesions and their internal and peripheral blood flow signals. MRI scanning (T1WI and T2WI scans) was performed on the pelvis to show the space-occupying lesions of the uterus, blood flow of the lesions, and relationship between the uterus and surrounding tissues and organs. Preoperative preparation mainly included routine skin preparation and intestinal preparation. Skin preparation was to prepare the skin and predict the degreasing and degassing of the skin area for the ultrasound beam to pass through (ultrasound beam path), and bowel preparation was mainly catharsis and bowel cleaning before treatment to avoid interference of gas on ultrasonic images and reduce the risk of intestinal injury.

In HIFU treatment, the prone position was used. Indwelling catheterization and appropriate filling of bladder (200–300 ml) were performed. Appropriate sedation and analgesia (fentanyl, midazolam and morphine) were used during the whole process of treatment. The patient's lower abdomen was immersed in degassed water, the treatment area was selected by a diagnostic ultrasound system, and the intestine and pubic symphysis were avoided in the ultrasound beam path. When the focus was fully exposed and the scope of adenomyosis was determined, the treatment scope was formulated. Each lesion was divided into several layers with a layer thickness of 5 mm, and the lesion was treated layer by layer from deep to shallow. The treatment parameters of the ultrasound were transmission frequency 0.8 MHz and input power 50–400 W (350 ± 30), and the temperature in the target tissue under these conditions would reach 60–100 °C (85 °C ± 6.3 °C), which was sufficient to necrotize the target tissue^12–16^. Point scanning was used and repeated 6–12 times, with each point irradiation as 1-2S and an interval of 1-3S. During the operation, the treatment power, position and treatment time were appropriately adjusted according to whether the patient had discomfort during the operation and whether the lesion could be efficiently coagulated or necrotized based on the real-time ultrasound monitoring. Firstly, the treatment power was tried from lower to upper values to find the appropriate value of power, and the time of treatment was also tried out. These treatment parameters like the power, treatment tine and position were different in different individuals. After an appropriate power was determined, the treatment time was also determined to range 1-2S to monitor the grey increase in the target area before manually moving the HIFU focus to the next point for ablation. The moving of the HIFU focus to the next point for ablation took about 1-3S which was the interval from one point to the next point for ablation. The total treatment time ranged 1–4 h (mean 148.8 min ± 86.9) according to the size of lesion. One month after HIFU treatment, the LN-IUS was inserted into the uterine for combined treatment in all patients.

Real-time monitor with a diagnostic ultrasound system.

**Figure 1:**
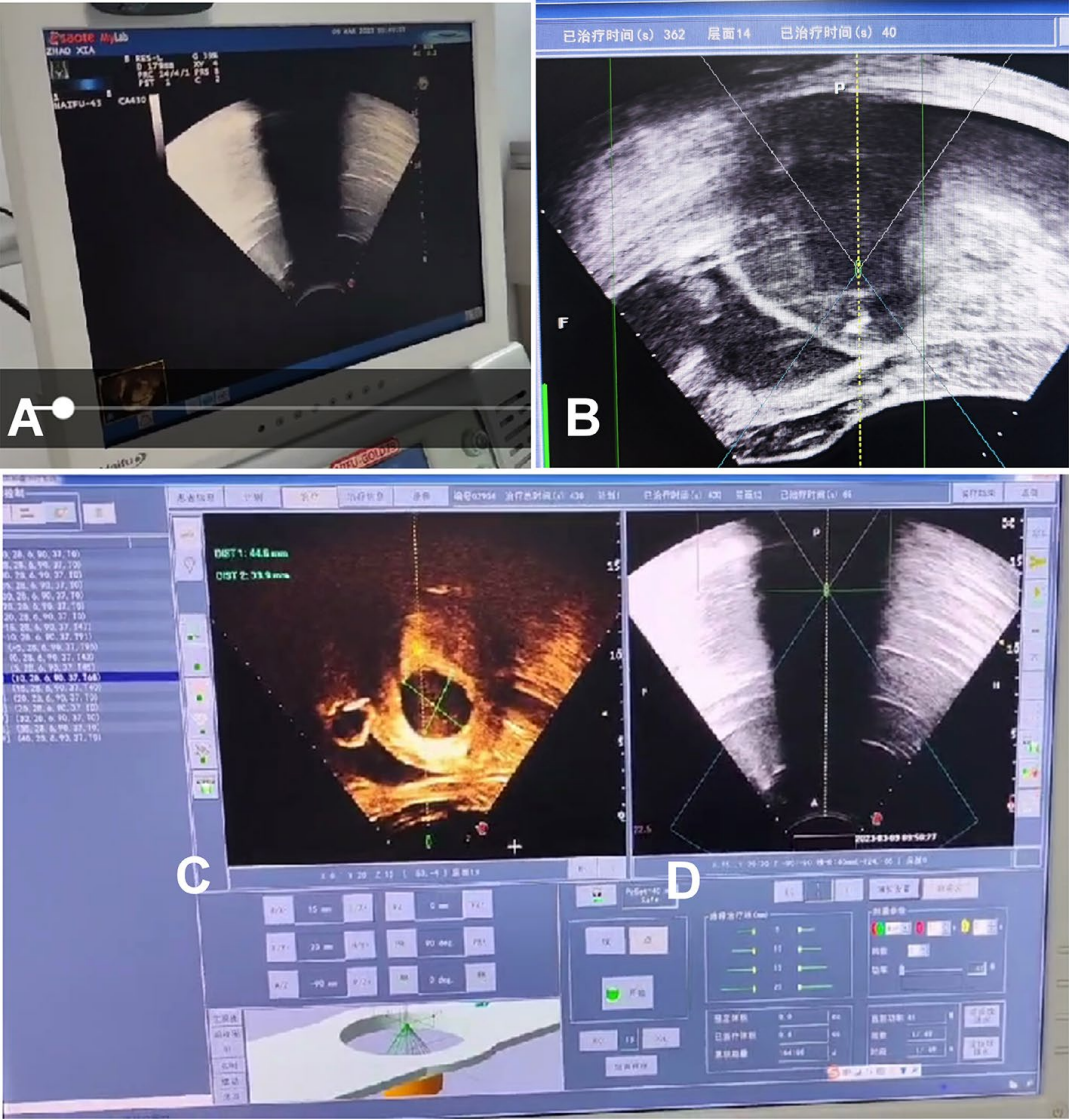
Real-time ultrasound monitoring of treatment of uterine ademomyosis with high-intensity focused ultrasound (HIFU). (**A**) Imaging of real-time ultrasound monitoring was displayed on the screen of one ultrasound scanner. (**B**) Real-time ultrasound monitoring showed that the total treatment time was 362 s, the treatment was on the 14th layer, and the treatment time on this layer was 40 s. (**C**) HIFU treatment imaging was displayed on the monitor screen of HIFU scanner. (**D**) The imaging of real-time ultrasound monitoring (**A**) was transferred to the HIFU screen (**D**) for real-time monitoring of the HIFU treatment effect, and if the treatment caused significant gray increase in the whole lesion area, the HIFU treatment would be stopped.

During the treatment, real-time monitor with a diagnostic ultrasound system was performed to observe the changes of lesion and surrounding normal tissues in real time so as to focus energy on the treatment area and avoid damaging surrounding tissues (Fig. 1). When there was an obvious gray increase or mass gray in the target area, coagulative necrosis in the treatment area could be confirmed, and treatment was then move to the next point until the whole area was completely treated. During treatment, if the patient had abdominal skin burning pain or lumbosacral, hip and lower limb pain, the irradiation treatment was stopped.

### Parameters for evaluation

Before and after treatment, the following parameters were evaluated for treatment effect: menstrual changes (menstrual cycle, menstruation period and volume), changes in hemoglobin levels, dysmenorrhea, changes of blood flow signal within the uterine lesion, volume of the uterine lesion, pregnancy, and adverse events. The menstruation volume was decided according to the number of tampons used in each menstruation period before and after HIFU, and the percentage change was calculated with the number of tampons after HIFU to compare with that before HIFU. The volume of uterine lesion was measured on T2WI images based on three-dimensional diameters: the longitudinal (D1), anteroposterior (D2) and transverse (D3) diameter, with the volume V = 0.5233 × D1 × D2 × D3^4^.

Blood flow signals within the uterine lesion were defined in line with the Adler blood supply grade in the uterine fibroids^25^: 0, no blood flow signal within the lesion; I, stellate blood flow signals less than 1 mm in diameter; II, short strip blood flow signals with the number of blood vessels ≤ 3; III, affluent blood flow within the lesion with > 4 blood vessels or vessels interwoven into a network in the uterine lesion.

Size reduction and blood flow signal decrease were used to evaluate the effect of combined treatment. The treatment effect was judged to be remarkable when the blood flow signals within the uterine lesion were significantly decreased to below grade I and the lesion volume was reduced > 50% that before treatment, moderate when the blood flow signals were decreased to below grade II with the lesion volume reduction > 25% that before treatment, and ineffective when the blood flow signals were still over grade III with the lesion volume decrease < 25% that before treatment. The effective rate was calculated as (number of remarkable effect + number of moderate effect)/total number of patients × 100%.

### Follow up

Follow-up was performed 1, 3 and 6 months after the procedure, including blood routine, ultrasound and MRI to observe the size of the lesion and blood supply. Improvement of clinical symptoms such as reduction of menstrual volume, change of cycle, anemia, dysmenorrhea and side effects after treatment were also recorded. The volume, internal blood flow echo and image changes of ablation focus after HIFU treatment were evaluated by transvaginal ultrasound and MRI. The size, volume and sonogram of uterine tumor focus were compared with those before treatment. The treatment effect and the effective and ineffective rate of treatment were evaluated.

### Statistical analysis

The statistical analysis was performed with the SPSS software (Version 18.0, IBM, Chicago, IL, USA). Measurement data were presented as mean ± standard deviation and tested with the t-test, and enumeration data were presented as numbers and percentages and tested with the Chi-square test. The significant *P* was set at < 0.05.

## Results

### Subjects

**Figure 2:**
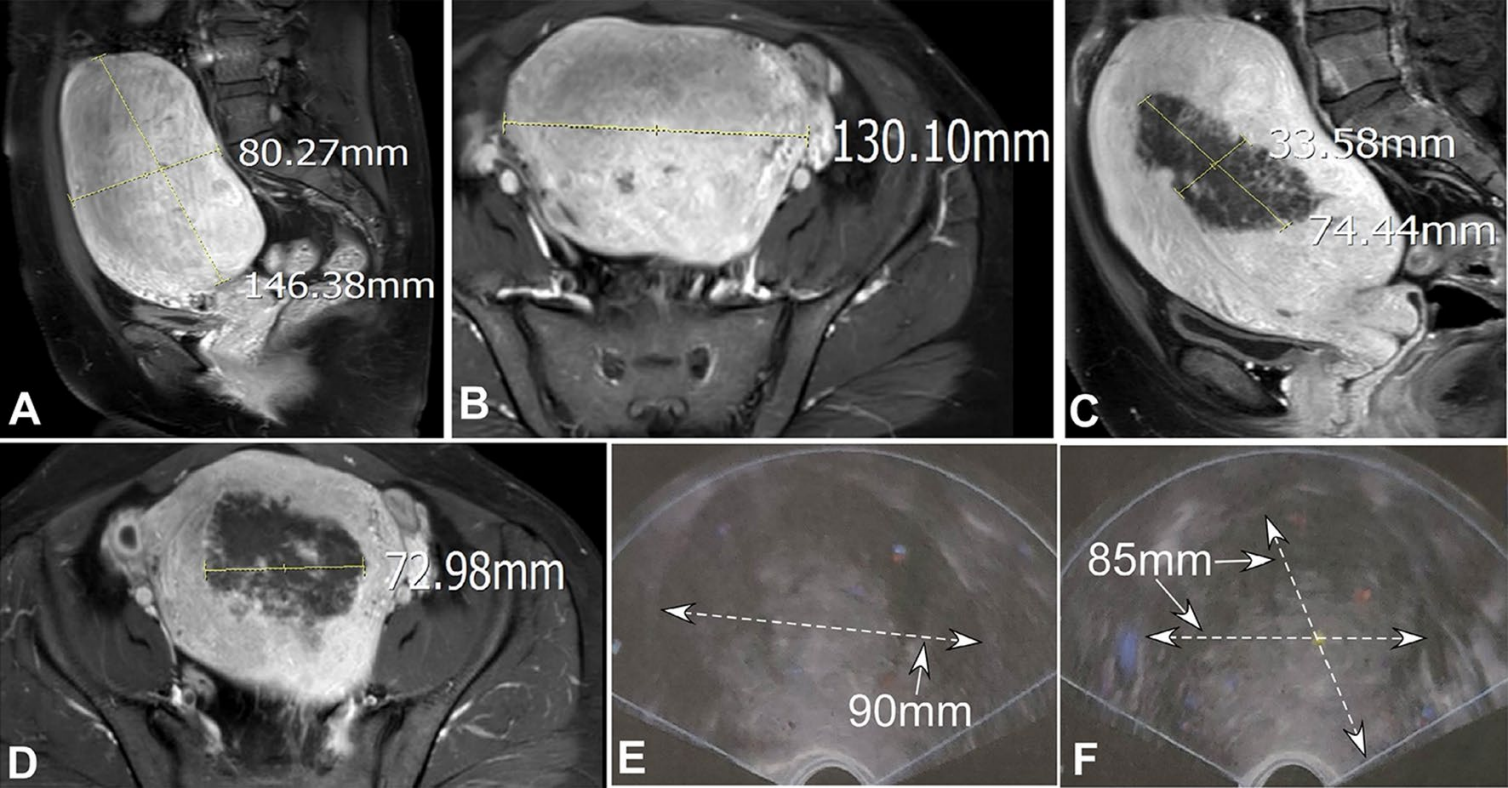
A female patient in their early 40 s with dysmenorrhea and increased menstrual volume for ten years was detected to have uterine adenomyosis. (**A**) and (**B**) On enhanced magnetic resonance imaging (MRI), the uterus measured 146.38 mm in the long diameter, 80.27 in the anteroposterior diameter (**A**) and 130.10 mm in the transverse diameter (**B**). (**C**) and (**D**) After HIFU treatment, the ablative area of the uterus was 74.44 mm in the long diameter, 33.58 mm in the anteroposterior diameter (**C**), and 72.98 mm in the transverse diameter (**D**). (**E**) and (**F**) Three months after treatment, the uterus was spherical in shape with the size of 85 mm × 9 mm × 85 mm (arrows), the posterior wall of uterus was diffusely thickened, with an unclear boundary, and the blood flow was not rich. The echo in the right fundus was uneven with an unclear boundary is unclear and no blood flow distribution.

**Figure 3:**
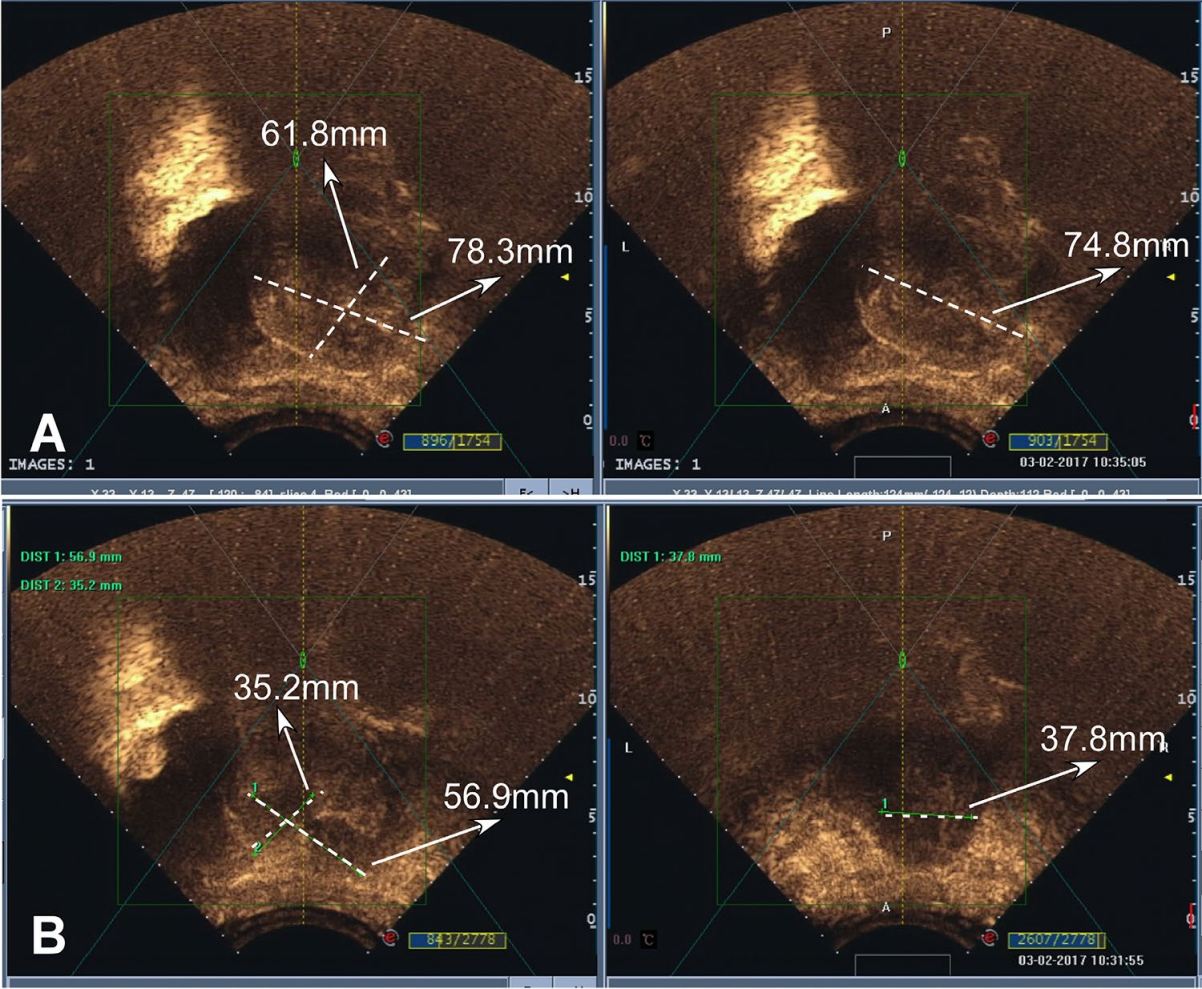
A woman in their 30 s had dysmenorrhea and increased menstrual volume and was found to have uterine adenomyosis. (**A**) Before high-intensity focused ultrasound (HIFU) treatment, the lesion measured 61.8 mm × 78.3 mm × 74.8 mm on ultrasound imaging. (**B**) After HIFU treatment, the lesion was reduced to 35.2 mm × 56.9 mm × 37.8 mm.

A total of 131 patients with adenomyosis aged 27–53 (mean 39.0 ± 5.2) years were enrolled to undergo the HIFU combined with the LN-IUS treatment (Figs. 2 and 3). The main presentations were increased menstrual volume (> 80 mL, n = 82), menstrual changes (n = 98), anemia (n = 65), dysmenorrhea (n = 78) and infertility (n = 6). The size of the lesion ranged 2.0 cm-10.5 cm (mean 5.3 ± 1.2) with a mean volume of 39.5 ± 3.6 mm^3^ and a mean time of treatment of 2.6 ± 0.6 (1–4) hours. HIFU treatment was technically successful in all patients (100%) without any difficulty or causing any complications.

### Volume of uterine lesions

Follow-up was performed in 129 (98.5%), 123 (93.9%) and 112 (85.5%) patients, respectively, 1, 3, and 6 months after the procedure. The volume of the uterine lesion was significantly decreased at follow-ups (Table 1). At one-month follow-up, 26 (20.2%) patients achieved remarkable effects with the lesion volume significantly decreased > 50% that before treatment, 67 (51.9%) moderate effects with the volume decreased > 25%, and 36 (27.9%) were ineffective with the volume decreased < 25%, with an effective rate of 72.1%. At three-month follow-up, 31 (25.2%) patients achieved remarkable effects, 72 (58.5%) moderate effects, and 20 (16.3%) ineffective, with an effective rate of 83.7%). At the last six-month follow-up, 32 (28.6%) patients achieved remarkable effects, 75 (67.0%) moderate effects, and five (4.5%) ineffective, with an effective rate of 95.5%.

**Table 1:** Changes of lesion volume at different time points in ultrasound.

Time for follow-up	No. of patients	Remarkable effect	Moderate effect	Ineffective	Effective rate
1 m	129 (98.5%)	26 (20.2%)	67 (51.9%)	36 (27.9%)	93 (72.1%)
3 m	123 (93.9%)	31 (25.2%)	72 (58.5%)	20 (16.3%)	103 (83.7%)
6 m	112 (85.5%)	32 (28.6%)	75 (67.0%)	5 (4.5%)	107 (95.5%)

Effective rate = (number of remarkable effect + number of moderate effect)/total number of patients × 100%.

### Blood flow signals

The blood flow signals within the uterine lesion were significantly reduced after treatment (Table 2). At one month after HIFU treatment, 32 (24.8%) patients achieved remarkable effects with the blood flow signals decreased to below grade I, 60 (46.5%) moderate effects with the blood flow signal decreased to below grade II, and 37 (28.7%) ineffective with the blood flow signal remaining over grade III, resulting in an effective rate of 71.3% in case of blood flow signal reduction. At three-month follow-up, 39 (31.7%) patients achieved remarkable effects, 72 (58.5%) moderate effects, and 12 (9.8%) ineffective, resulting in an effective rate of 90.2%. Six months later, 39 (34.8%) patients achieved remarkable effects, 65 (58.0%) moderate effects, and eight (7.1%) ineffective, leading to an effective rate of 92.9%.

**Table 2:** Changes of blood flow signals within the lesion in ultrasound.

Time for follow-up	No. of patients	Remarkable effect	Moderate effect	Ineffective	Effective rate
1 m	129 (98.5%)	32 (24.8%)	60 (46.5%)	37 (28.7%)	92 (71.3%)
3 m	123 (93.9%)	39 (31.7%)	72 (58.5%)	12 (9.8%)	111 (90.2%)
6 m	112 (85.5%)	39 (34.8%)	65 (58.0%)	8 (7.1%)	104 (92.9%)

Effective rate = (number of remarkable effect + number of moderate effect)/total number of patients × 100%.

### Changes in clinical presentations

At the last six-month follow-up, the menstrual cycle was significantly (*P* < 0.05) decreased from 31.4 ± 3.5 days before treatment to 28.6 ± 1.9 days six months later, the menstrual period was significantly shortened from 7.9 ± 1.2 days before HIFU to 6.5 ± 1.3 days, and the menstrual volume was significantly (*P* < 0.05) decreased from 100 to 49% ± 13% (Table 3). The serum hemoglobin was significantly (*P* < 0.05) increased from 90.8 ± 6.2 g/L before HIFU treatment to 121.6 ± 10.8 g/L at six-month follow-up for patients with anemia.

**Table 3:** Menstrual changes before and after HIFU treatment.

Variables	Before HIFU	After HIFU	P
Case no	131	112	
Menstrual cycle (d)	31.4 ± 3.5	28.6 ± 1.9	0.04
Menstrual period (d)	7.9 ± 1.2	6.5 ± 1.3	0.03
Menstrual volume (%)	100% ± 0	49% ± 13%	0.001

Among 78 patients with dysmenorrhea, dysmenorrhea disappeared completely in 45 (57.7%) patients, and twenty-seven (34.6%) patients experienced significantly relieved dysmenorrhea three months after the HIFU treatment. Among seventy-two (92.3%) patients who finished the six-month follow-up, sixty-five (90.3%) patients had the dysmenorrhea completely relieved, and the other seven (9.7%) patients had only slight dysmenorrhea which did not affect their daily life.

Three months after treatment, five (83.3% or 5/6) cases obtained intrauterine pregnancy, and six months after treatment, all six patients experienced intrauterine pregnancy.

### Adverse events

Among 131 patients treated with HIFU, no skin burns occurred in 129 (98.5%) patients, and two (1.5%) patients had a burn of 1 × 2 cm in the treatment area which was healed after appropriate treatment. The skin in 4 (3.1%) patients was slightly swollen with slightly increased skin temperature, and the swelling subsided after intermittent ice compress. One to seven days after HIFU treatment, 15 (11.5%) patients had mild lower abdominal pain and low fever, which were improved after symptomatic treatment. Subcutaneous induration occurred in 3 (2.3%) patients, including 2 patients with pain. The pain disappeared after 3–5 days of oral non-steroidal anti-inflammatory drugs, and the subcutaneous induration became smaller and disappeared after 1–2 months. One month after treatment, 5 patients had vaginal discharge after menstruation, which was colorless, transparent and tasteless. The menstruation was returned to normal after 1–2 menstrual cycles.

Three months after treatment, dysmenorrhea in one patient with adenomyosis was not remarkably improved, and the menstrual volume did not decrease significantly. Hysterectomy was performed in this patient as required. Six months after treatment, no other serious side effects occurred in all follow-up patients. In total, adverse events occurred in 24 (18.3%) patients without causing severe consequences.

## Discussion

In this study, the effect of HIFU combined with the LN-IUS treatment was investigated on adenomyosis, and it was found that the HIFU combined with the LN-IUS treatment was effective and safe with most patients achieving remarkable and moderate effects even though the long-term effects remained to be explored. Currently, our team was still working on the follow-up of these patients so as to get a better picture of the long-term HIFU treatment effect.

HIFU treatment of uterine adenomyosis is to focus ultrasound on the target area in the organ from outside the body to form a focal area where high-intensity ultrasound converges. The biological effect of high-intensity ultrasound causes degeneration and coagulative necrosis of focal tissue cells in the focal area so that the volume of uterine lesions is reduced and the clinical symptoms are relieved^1–5,7^. During the HIFU treatment procedure, when the local temperature in the lesion reached 60–100 ℃, the lesion protein could be coagulated and necrotized, thus decreasing the lesion size and achieving the HIFU treatment effect^12–16^. Because of blood flow in the lesion, some thermal energy will be taken away during HIFU treatment, and thus, the local lesion temperature seems high for destruction of the lesion. Moreover, during the treatment process of HIFU, the treatment focus and surrounding normal tissues and organs are also monitored in real time through a diagnostic ultrasound system for real-time monitoring, which is to observe the effect of uterine lesion destruction and protection of surrounding tissues and organs. HIFU can result in effective destruction of the uterine lesion and rapid recovery with a low risk of complications^4^. Currently, there are two modalities to guide HIFU treatment: MRI and ultrasound which may be integrated with HIFU. Ultrasound imaging is currently the most prominent in this application because of its ease of use, cost-effectiveness, and easy accessibility to the uterine lesions like the adenomyosis or adenomyoma^1–5,7,26–28^. Ultrasound-guided HIFU has also been used in other benign and malignant cancers like symptomatic benign thyroid nodules, colorectal cancer liver metastasis, prostate cancer, advanced cervical cancer, advanced pancreatic cancer, desmoid tumors, and breast cancer^29–37^. However, the use of MRI as a modality of guidance has been on the rise with satisfactory results^3,38–41^. Moreover, the signal intensity on T2WI and enhancement on T1WI can be used as factors affecting the HIFU ablation effect. Yang et al.^4^ studied factors affecting HIFU treatment effect and found that the signal intensity on T2WI was the most important factor affecting the effect of HIFU ablation, followed by enhancement type on T1WI, uterine anteriorposterior diameter, and platelet count.

In evaluation of the treatment effect of HIFU on uterine fibroids, the non-perfused volume ratio (NPVR) had been used, with the NPVR being equal to non-perfused volume to the whole volume of lesion. It has been reported that NPVR was the gold standard for assessing the effect of HIFU ablation, with a NPVR of over 80% as sufficient ablation^4^. It has been reported that NPVR was a good method to assess the effect of HIFU ablation, with a NPVR of over 80% indicating sufficient ablation^4^. In a study of NPVR ≥ 80% for predicting HIFU ablation for uterine fibroids in 1000 patients with 1000 uterine fibroids^4^, 758 patients had achieved sufficient ablation while 242 achieved only partial ablation, with the median NPVR of 88.3% (interquartile range: 80.3–94.8%). Zhou et al.^5^ studied ultrasound-guided HIFU for devascularization of uterine fibroids and found a NPVR of 79.91% ± 38.25% (ranging 0.56–100%) at one month and 75.23% ± 34.77% (ranging 0–100%) at six months after HIFU treatment. Moreover, a negative correlation existed between the NPVR and Adler grade of blood flow signal^5^. The non-perfused volume of uterine fibroids can be used to assess the HIFU treatment effect, and the NPVR has been suggested to be over 50% for effective treatment with sufficient fibroid shrinkage and symptom alleviation^5,42^.

The Adler grade of blood flow signal has been widely applied by ultrasound practitioners to semi-quantitatively assess blood flow within a tumor lesion^43,44^. After investigating the correlation between color power Doppler sonographic measurement of breast tumor vasculature and immunohistochemical analysis of microvessel density for the quantitation of angiogenesis, Yang et al.^44^ found that higher microvessel density was noted in malignant than benign breast masses (*P* < 0.0005) and that tumor vessel numbers were in a significant positive correlation with tumor size (*P* < 0.05) and progesterone receptor negativity (*P* < 0.05). Moreover, a significant positive correlation was detected between microvessel density and the number of intratumoral blood vessels assessed in color power Doppler sonography (*P* < 0.05). After studying the relationship of Adler grade by color Doppler flow imaging and the clinical pathological parameters of cervical cancer, Che et al.^43^ found that the Adler grade of blood flow signals was positively correlated with the clinical stage, pathological type and squamous cell carcinoma subtypes of cervical cancer (all *P* < 0.05), suggesting that Adler grades are closely associated with the clinical pathology of cervical cancer as an effective and convenient way for assisting evaluation of cervical cancer. In a study of HIFU for devascularization of uterine fibroids by Zhou et al.^5^, the Adler grade of blood flow signal was decreased to grade 0 after HIFU treatment, and a significant (*P* < 0.05) negative correlation was detected in the Adler grade of blood flow signal with either shrinkage of fibroid volume or NPVR. Even though the Adler grade may be decreased to 0, there is still a possibility of small vessels existing in the treated fibroids, resulting in possible recurrence and regrowth of the fibroids. Contrast-enhanced ultrasound can be performed to increase the diagnostic accuracy of the Adler grade and sensitivity of micro-circulation detection so as to increase the degree of devascularization and subsequently the NPVR by the HIFU treatment^5,45^.

In our study, we used size reduction and blood flow signal decrease to assess the treatment effect of HIFU combined with the LN-IUS on the uterine lesion. With the size decrease of the uterine lesion, the blood flow signals would also decrease because HIFU can use its high energy to destroy tumor vessels and other tissues within the uterine lesion. Devascularization of the uterine lesion is able to prevent the growth and alleviate clinical symptoms, thus achieving the treatment goal. After HIFU treatment, the volume of the uterine lesion and the blood flow signals within the lesion were both significantly reduced at different follow-up points after treatment. At the 6-month follow-up, the effective rate reached over 90% for both size reduction and blood flow decrease. Moreover, with the decrease in the size and blood flow signals within the uterine lesion after HIFU treatment, the clinical symptoms were significantly alleviated and improved. Some infertile patients had regained fertility. The outcome of our study was comparable to those of previous studies which achieved symptom improvement and fibroid shrinkage in over 90% of patients^5,46–48^.

In comparison with other minimally-invasive approaches of treatment like UAE and radiofrequency ablation, HIFU treatment has decreased rates of vascular adverse events and relevant complications^4,5,7,10,18,26–28^. UAE may have impaired ovarian blood flow causing subsequently ovarian failure and infection leading to subsequent fallopian tube damage and fertility besides other complications like vaginal discharge, fever, and post-embolization syndrome^49^. The complications of HIFU may primarily include skin burns, abdominal pain and repeated interventions, with vascular adverse events being rarely reported, and only one patient was reported to suffer from deep venous thrombosis^50^. These complications can be easily managed in a short period of time^5^. Common adverse events in HIFU treatment involve skin burn, pain, fever, nausea, vomiting, skin blisters and hematuria^51^. Thermal injury to the skin-fat-muscle interface is possible because fat had high efficiency of absorbing energy. Pelvic pain may result from inflammation, and vessel occlusion may cause pain. Abnormal vaginal discharge may be caused by the effect of HIFU on the endometrium. However, all these adverse events are not severe and can be easily managed. In our study, adverse events occurred in 24 (18.3%) patients without causing severe consequences.

Besides HIFU ablation of the uterine lesion, the LN-IUS was used to enhance the treatment effect one month after the HIFU ablation. The LN-IUS contains progesterone and can slowly and constantly release progesterone to decrease the amount of menstrual blood and relieve the pain during the menstruation period^52,53^. It is a reversible, effective, and long-term therapy for adenomyosis, and many studies have shown that it can effectively decrease menstrual bleeding, dysmenorrhea and uterine volume, with an overall satisfaction rate of 72%^54–56^. It has been reported that use of this device after uterine surgery is able to decrease the recurrence of adenomyosis^57^. In this study, the LN-IUS was used to enhance the effect of HIFU treatment, with good effects on the lesion volume reduction, blood flow signal decrease, and symptom improvement.

Some limitations existed in this study, including the retrospective and one-center study design, a small cohort of patients, Chinese patients enrolled only, and no control, which may all affect the generalization of the outcome. Explanation of the outcome should be cautious. Moreover, we did not evaluate the non-perfused volume ratio of uterine lesions after HIFU treatment which may be used as an imaging standard for assessing the HIFU effect^4,39,58^. All these issues may produce some publication bias to affect the generalization of this study. Future prospective, multicenter, controlled, and randomized studies with diverse populations of race, ethnicity and culture background and long-term follow-up outcomes will have to overcome these issues for better outcomes.

In conclusion, HIFU combined with the LN-IUS can be safely and effectively used to treat adenomyosis with good effects in the short term even though long-term effects remain to be confirmed.

## Data Availability

Data are available from the corresponding author on reasonable request.
